# Hydatidiform mole: A sour encounter with a grapy case

**DOI:** 10.4103/0019-5049.79900

**Published:** 2011

**Authors:** Madhuri S Kurdi

**Affiliations:** Department of Anaesthesiology, Karnataka Institute of Medical Sciences, Hubli, Karnataka, India

**Keywords:** Anaesthesia, cardiopulmonary distress, complications, critical, hydatidiform mole, hyperthyroidism, mortality, suction evacuation, tachycardia, techniques

## Abstract

Hydatidiform mole cases are usually uncomplicated. However, few cases can be associated with perioperative complications of a critical nature, which can lead to substantial mortality and morbidity. Here is a report of one such case, which, in spite of extensive perioperative management, led to mortality.

## INTRODUCTION

Molar pregnancy is common in Oriental countries — Philippines, China, Indonesia, Japan and India, as also in Africa and Central and Latin America. The incidence in India is one in 400;[1] 80% of the cases are uncomplicated and 20% are associated with an extensive list of perioperative complications, some of which may be of a critical nature.[2] Prevalence of hyperthyroidism during complete molar pregnancy is as high as 7%.[3] We report, here, a case of molar pregnancy with hyperthyroidism, wherein the patient developed acute cardiopulmonary distress following suction evacuation under general anaesthesia.

## CASE REPORT

A moderately built 25-year-old female, with a 24-week molar pregnancy was brought to the operation theatre in the wee hours of the morning for emergency suction-evacuation. Her medical and surgical histories were unremarkable. She was highly irritable, afebrile and moderately pale. She had a pulse rate of 156 beats / minute, blood pressure of 110/80 mm Hg, respiratory rate of 32 breaths / minute and mild pedal oedema. The other general and systemic examinations were unremarkable. Her haemoglobin level was 8 g%. Her thyroid function tests were markedly deranged showing severe biochemical hyperthyroidism [T3 =- 6.46 nmol/L, T4 = 470 n mol/L and TSH = 0.03 μIU/mL]. The serum βhCG levels were markedly raised. An ultrasonogram showed signs of a complete molar pregnancy.

Pre-induction, the patient was given intravenous (IV) dexamethasone 2 mg, IV Fentanyl 100 μg and IV Metaprolol 0.5 mg. The pulse rate came down to 100 beats / minute. Rapid sequence induction was done with Inj. Thiopentone 250 mg and IV Succinyl choline 80 mg. A cuffed oral endotracheal tube No. 7 was passed. She was maintained on oxygen and nitrous oxide on intermittent positive pressure ventilation, with Vecuronium as a muscle relaxant. Intraoperatively, the pulse rate was maintained at around 100 beats/ minute, blood pressure at 110 / 60 mm Hg and oxygen saturation (Spo_2_) at 99%. The surgical procedure lasted for 15 minutes. The total intraoperative blood loss was approximately 300 mL. Ringers Lactate of 500 ml was infused intraoperatively. One unit of blood was started. She was extubated after reversal. On the table, she appeared slightly restless and mildly tachypnoeic with SpO_2_ 98% on room air. She was awake. Her chest was clear. She was shifted to the postoperative ward. Within half an hour, fine crepitations developed in her chest. She was given Inj. Furosemide 60 mg and was shifted to the Intensive Care Unit. A central venous pressure (CVP) line was inserted. The CVP was 10 cm of water. Slowly, over the next six hours, she became a lot more tachypnoeic. The crepitationsincreased. Blood pressure was stable. She was intubated and put on mechanical ventilation with positive end-expiratory pressure (PEEP). She was nursed in a propped-up position. As per the physician’s advice, she was put on Inj. Metaprolol 5 mg OD, Tab Carbimazole 20 mg bd and Inj. Furosemide 40 mg bd. The next day, her condition remained the same. Urine output was maintained. She was tachypnoeic. Haemoglobin was 7.6 g%. A chest X-ray revealed bilateral fluffy opacities with normal cardiac size, suggestive of pulmonary oedema. An electrocardiogram revealed tachycardia. Arterial blood gas analysis revealed a low pO_2_. She was unable to maintain SpO_2_even on an fraction of inspired oxygen (Fio_2_) of 100% and PEEP. Thyroid function tests done 24 hours postoperatively showed improvement, but they were still deranged (T3 = 5.3n mol / L, T4 = 260 n mol /L and TSH = 0.05 μ IU/mL). The tachycardia persisted. Her blood pressure fell. Except for tachycardia, there were no other signs of high cardiac output. Dopamine and dobutamine infusions were started. Her condition worsened. In spite of extensive resuscitation efforts tried by us according to the advanced cardiac life support (ACLS) guidelines, she died 36 hours after evacuation of the mole.

## DISCUSSION

Anaemia, hyperthyroidism and acute cardiopulmonary distress are significant complications of complete molar pregnancy.[2,4] This case was associated with all these complications.

Anaemia in molar pregnancy is secondary to chronic occult per vaginal bleeding and from massive blood loss during surgery.[2] Severe anaemia can lead to left ventricular failure and pulmonary congestion, leading to cardiopulmonary distress.[4] However, this case had only a moderate degree of anaemia. Inadvertent fluid overload could occur during a central neuraxial block, in an attempt to sustain the blood pressure. This can be a cause of acute cardiopulmonary distress.[2,4] However, the I.V fluids were restricted and CVP was monitored. Hence, the occurrence of inadvertent fluid overload in this case was unlikely.

Hyperthyroidism in molar pregnancy is attributed to excess hCG, which has a weak thyroid stimulating activity.[3] This trophoblastic hyperthyroidism may be biochemical (50% cases) or clinical (5% cases).[5,6] The hyperthyroidism may be controlled by evacuation of the molar tissue.[3] Pre-operatively, the patient should be put on anti-thyroid drugs and if there is no time to make her pharmacologically euthyroid, iodine and β-blockers should be administered.[7] We used I.V metoprolol and dexamethasone, which were the only drugs available to us at the time of induction. There was not enough time to make her pharmacologically euthyroid, because of the sudden increase in per vaginal bleeding, leading to an urgent need to take up the case for evacuation.

Pulmonary hypertension, which is correlated with inadequately controlled hyperthyroidism can result in heart failure and pulmonary oedema.[8] This is a possibility in our case. Few authors have reported incidences of acute respiratory distress syndrome (ARDS) and pulmonary oedema in cases of vesicular mole with hyperthyroidism.[7,9]

Acute cardiopulmonary distress has been observed after evacuation of molar pregnancy in 27% of the cases and especially in patients with a uterine size of 16 weeks or greater.[4] It usually develops within 12 hours of evacuation. It is marked by cough, tachycardia, tachypnoea, hypoxemia, diffuse rales and bilateral pulmonary infiltrates, on a chest radiograph. Trophoblastic embolisation is the proven etiology in more than half of these cases.[4] Variable amounts of trophoblastic cells escape from the uterus into the venous outflow at the time of evacuation.[10] The clinical signs vary in severity and some fatalities have been described.[2,10] The possibility of trophoblastic embolisation being a cause of the cardiopulmonary distress cannot be ruled out in this case.

Transfusion-related acute lung injury, although rare, is another possible cause of cardiopulmonary distress. It occurs within six hours of transfusion and is marked by non-cardiogenic oedema. The patient usually recovers within 96 hours.[11] Identification of neutrophil antibodies could help in its diagnosis, but it was not possible in our setup. It was a diagnosis of exclusion. Our patient did not recover despite receiving optimal ventilatory and inotropic support, in addition to anti-thyroid medication and intensive care.

Sedation, monitored anaesthesia care, total intravenous anaesthesia, general anaesthesia (GA) and spinal anaesthesia (SA) are the various anaesthesia techniques that can be adopted for a case of evacuation of molar pregnancy.[2,4,6] GA was used here, as it provided haemodynamic stability. It is useful for the patient who is losing a large amount of blood.[2,3] A successful spinal anaesthesia technique has also been reported to have been used for the evacuation procedure in a case of vesicular mole with hyperthyroidism.[3,4,12] The advantages included preferable effects on the pulmonary system, ease of technique, and favorable nontocolytic pharmacological properties.[12]

## CONCLUSIONS

Every anaesthesiologist should be well aware of the critical nature of perioperative complications that can be associated with a molar pregnancy. A detailed pre-anaesthesia work-up, pre-operative optimisation of the patient’s thyroid and volume status, planning and conducting anaesthesia carefully and being prepared for advanced perioperative anaesthetic management are a must for any case of molar pregnancy.
